# Mobile Money and the importance of timely, complete payments to frontline health campaign workers in the fight to eradicate polio: pilot experience from a World Health Organization digital payment platform in Africa

**DOI:** 10.1186/s12913-022-08990-4

**Published:** 2023-01-07

**Authors:** Ahmed Hamani, Idil Hussein Jama, Mian Amoakon Yves Roland, Leah Wanjeri, Abena Aboagyewaa Oppon-Kusi, Dorcas Karimi, Patsy Kiconco, Oromena Edwin Akpotu, Mahafous Saka

**Affiliations:** 1 World Health Organization–Regional Office for Africa, Cité du Djoué, PO Box 06, Brazzaville, Republic of Congo; 2 World Health Organization, 351 Francis Baard St, Metropark Building, 7th Fl, PO Box 13113, Pretoria, South Africa

**Keywords:** Africa, Cash-based payments, Côte d’Ivoire, Frontline workers, Immunization programs, Mobile Money, poliomyelitis eradication, Poliovirus vaccines, Vaccination campaign

## Abstract

**Background:**

In response to the increase in vaccine-derived poliovirus type 2 in Côte d’Ivoire, Mali, and many other African countries from 2017 to 2019, concentrated efforts are needed to improve the effectiveness of vaccination campaigns. Frontline polio health campaign worker engagement and job retention are critical to successful campaign implementation, as well as timely, in-full payment to these workers via an electronic system.

**Methods:**

The Global Polio Eradication Initiative and its partners designed a road map to implement the World Health Organization Mobile Money digital payment system for health campaign workers across designated African Region countries and country-specific areas. The road map included: (1) strategy communication about Mobile Money to key stakeholders; (2) prioritization of Mobile Money pilot countries; (3) establishment of a digital finance team to support Mobile Money rollout for polio campaigns; (4) implementation of Mobile Money in select pilot areas; and (5) documentation by the digital finance team of Mobile Money implementation across pilot areas. At the country-specific level, and as described in the first pilot campaign in Côte d’Ivoire, implementation of Mobile Money occurred in 3 phases: precampaign, campaign, and postcampaign.

**Results:**

Mobile Money was piloted in Côte d’Ivoire, Democratic Republic of the Congo, Ghana, Mali, and Republic of the Congo. Although program reach varied by country, the percentages of payments successfully made via Mobile Money in pilot countries were high: In campaign round 1, 99% of campaign workers in 2 regions in Mali, and 99% of campaign workers in 5 districts in Ghana were paid successfully. In Cote d’Ivoire, Mobile Money was piloted in all 113 districts for campaign rounds 1, 2 and 3, and in 4 districts in Abidjan for campaign round 3. In rounds 1, 2 and 3, 99.6%, 99.6%, and 99.9% of payments to polio health campaign workers, respectively, were made successfully.

**Conclusion:**

Implementation of the Mobile Money pilot program, particularly across Côte d’Ivoire, demonstrates the value of an electronic payment system in addressing frontline polio health campaign worker need for timely, in-full payment. The World Health Organization-led Mobile Money pilot program can serve as a model for agencies committed to delivering greater efficiencies and improved health campaigns in resource-challenged settings.

## Background

In many African countries, the burden of circulating vaccine-derived poliovirus type 2 (cVDVP2) increased from 2017 to 2019 [1]. Within this time frame, the period July 2019–February 2020 was marked by particularly pronounced viral circulation with polio outbreaks occurring in, but not exclusive to, Mali, Ghana, Côte d’Ivoire, Democratic Republic of the Congo (DRC), Ghana, and Mali [2]. In response to this unanticipated emergence of cVDPV2, the Global Polio Eradication Initiative (GPEI) established a full-time rapid response team (RRT) in Africa to work in partnership with the World Health Organization (WHO) and the countries’ respective ministries of health. Officially located in the WHO Regional Office for Africa (AFRO), the “virtual first response” team led several critical efforts designed to stop the spread of polio. The rapid response team’s work included boosting ongoing cVDPV2 outbreak response activities, enhanced acute flaccid paralysis surveillance, and building individual country capacity in the use of innovations such as smart phones to enhance polio eradication.

### The importance of timely, complete payments to frontline polio health campaign workers

In working to stop polio outbreaks in the region, the GPEI recognized a significant opportunity to improve polio campaign implementation: ample evidence shows that disruptions in routine immunization largely stem from lack of incentives to frontline health workers who implement vaccination campaigns [3–5]. These workers may include door-to-door vaccination campaigners, vaccinators, supervisors overseeing campaign timelines, and others who are instrumental in campaign execution. When frontline polio health campaign workers are not paid in a timely manner or completely, there are negative consequences for the workers themselves, as well as serious negative effects on campaign worker retention [3, 6–8]. Challenges related to payment and worker retention affect the success of polio vaccination campaigns.

In a study conducted prior to 2004, researchers found that the success of Nigeria’s polio eradication initiative depended on timely, direct, and full payment of vaccination personnel [7]. Following the establishment of a Mobile Money payment system for frontline workers, challenges with making timely, direct, and full payments to workers were overcome. Although operational barriers within the polio vaccination campaign remained, management of payment issues was critical to advancing the 2013–2018 polio endgame strategic action plan.

A more recent case study that examined managerial and operational hindrances to polio eradication in Katlang, Pakistan demonstrated the value of worker dissatisfaction with salaries and timely payment to campaign workers. In this case, both worker dissatisfaction with salaries and on-time delivery of salaries were significantly associated with a successful polio eradication process [8]. Issues associated with payment of frontline workers were found to hinder proper vaccination and immunization, leading to lack of polio eradication in Pakistan.

Similarly, a 2020 study that examined polio vaccination health campaigns in DRC and Ethiopia from 1988 to 2019 showed that payment issues affected campaign progress [6]. Based on data from an online survey and key informant interviews, study authors found DRC and Ethiopia encountered payment challenges including lack of payment to workers, lack of financial motivation among workers, and inappropriate diversion of financial resources. While the direct effect of these payment issues on health outcomes was not measured in this or other studies, according to the WHO, late and incomplete payments are associated with negative effects on frontline worker morale and satisfaction [9, 10]. This, in turn, results in decreased worker motivation and attendance, and makes polio health campaign worker retention challenging [3]. Indeed, in both DRC and Ethiopia, health worker shortages and high worker turnover, along with payment challenges, were linked to slowed campaign progress, failure to vaccinate people, and ultimately increased polio-associated health risks [6].

The literature supports the critical role of frontline health workers, noting that motivation of frontline workers is directly related to polio health campaign coverage and that frontline health campaign workers are key to ensuring that vaccinations are delivered [11]. Without strong routine immunization and timely vaccination campaigns in response to information from environmental surveillance data, polio outbreaks can and do occur [12].

## Assessment of specific gaps in the World Health Organization African region cash-based payment system

Following the widespread breakdown in regional polio campaign quality and timeliness from 2017 to 2020, WHO AFRO, along with GPEI and country ministries of health, recognized that outbreaks in a growing number of countries required a faster, more agile payment approach to ensure successful delivery of polio control campaigns. Postcampaign assessments identified the 3 main challenges with cash-based payments (which was standard practice in the pilot countries at that time). The first challenge was delayed disbursement of funds to operational levels. These delays resulted from issues with bank transfers or cash out at local banks and could result in payment delays as long as 1 month. Cash-based payments were associated with a second challenge: lack of financial transparency, which resulted in leakages and inefficiencies. Finally, cash-based payments were associated with numerous layers of fund transfer, which resulted in bureaucracy and duplicate processes.

To address these challenges, WHO AFRO in conjunction with the Bill & Melinda Gates Foundation (Seattle, Washington, USA) assessed the feasibility and potential of advancing Mobile Money throughout sub-Saharan Africa. An analysis of information from the Global Findex database, World Bank, and Global System for Mobile Communications Association found that use of Mobile Money in this region was possible, and could help address the challenges associated with cash-based payments. Additionally, in 2018, the WHO Global Strategy on Digital Health recognized the potential of digital technologies like Mobile Money to support health and prevent disease; this mandate for WHO member states helped prioritize the development and use of digital technologies like Mobile Money [13].

In response, WHO AFRO designed and implemented a digital payment system that would reduce lag time and ensure timely payment of funds to polio campaign frontline workers. WHO AFRO promoted greater efficiency by ensuring direct payment of campaign workers with little to no leakage of funds.

### Identification and rationale for the Mobile Money solution

The introduction of Mobile Money in select AFRO countries and country-specific areas was identified as a targeted solution to address challenges with cash-based payment systems. Mobile Money is a digital payment platform that allows people to receive, store, and spend money using a mobile phone [14]. Mobile Money technology is installed in the subscriber identity module card of a regular telephone or smartphone so users can access their money without being connected to a formal banking system. Notably, this platform differs from mobile banking in which users connect using Internet-enabled mobile devices to manage funds in their bank accounts.

Currently, sub-Saharan Africa is the global leader in use of Mobile Money; its 469 million registered Mobile Money user accounts comprise almost 50% of total Mobile Money users [15]. In all, 21% of sub-Saharan adults use Mobile Money and the subcontinent is home to all 10 economies worldwide in which more adults have a Mobile Money account than a traditional bank account. For example, > 30% of adults in Côte d’Ivoire and Senegal, and 40% in Gabon have a Mobile Money account.

Moreover, Mobile Money has 7 times more reach than automated teller machines and 20 times more than bank branches [16]. According to the World Bank, digitizing routine cash payments like wage payments could help dramatically reduce the number of unbanked adults—or adults who do not have access to a financial institution [15]. Many of these adults have the basic technology needed to receive payments digitally. For example, of the 60 million unbanked adults worldwide who receive government transfers in cash, two-thirds have a mobile phone.

This paper describes the implementation and rollout of the WHO Mobile Money payment system for polio health campaign workers across African region countries and country-specific areas. The focus is largely on Côte d’Ivoire, which was the first country to pilot Mobile Money on a national scale. Information and data from country-specific areas in Ghana and Mali are also provided.

## Methods

### Design and implementation

In 2020, the WHO AFRO developed a 5-step road map for rolling out the WHO’s Mobile Money digital payment system across designated AFRO countries and country-specific areas. In the first step—“strategy communication”—the AFRO Regional Director sent letters to WHO country offices and country ministries of health informing them of the switch to digital payments. Step 2 called for “country prioritization” in which the AFRO and Bill & Melinda Gates Foundation selected 8 polio priority countries or country-specific regions for implementation of digital payment solutions based on assessment of the areas’ digital economies, as well as their stakeholders’ receptivity to the program. During the third Mobile Money program rollout step—“Mobile Money pilot design”—the AFRO Digital Finance Team (DFT) was established to support the rollout of Mobile Money for polio campaigns, and developed an approach for the pilot design in Côte d’Ivoire, Ghana, and Mali. Step 4—“pilot implementation”—included various regions and districts in the pilot countries, and the digital payment system was adapted to suit the available technologies and capabilities in each country. In the fifth and final step—“documentation”—the DFT documented the processes of Mobile Money implementation during campaigns and 60 Decibels (New York, New York, USA), an impact measurement company, was engaged to survey campaign workers in Côte d’Ivoire and other pilot countries to determine: (1) the impact of switching to digital payments on campaign worker satisfaction; (2) how the digital payment system could be improved in the future; and (3) how many workers were digitally enabled during the campaign. The 60 Decibels postcampaign survey was administered via telephone interview to ~ 500 frontline campaign workers in each country. To control for recall bias, interviews were conducted 15–20 days postcampaign and no more than 6 weeks postcampaign. A random sample of all frontline campaign workers included on the campaign attendance registers was contacted.

During pilot implementation, the DFT identified key components of the digital payment system. These components were: (1) development of a polio health campaign worker database; (2) engagement of digital payment service providers and respective ministries of health to obtain buy-in for the project; (3) identification of bottlenecks and development of solutions for funds flow; (4) health campaign worker activity reporting; (5) payment to health campaign workers; (6) fielding of surveys; and (7) development of an exit strategy to perform a full database and Mobile Money wallet handover at project closure to the respective ministries of health so they can execute the payments independently. Following pilot implementation, Mobile Money expanded to DRC and Republic of the Congo later in 2020.

### Implementation of Mobile Money

To encourage broad program support and demonstrate program success, Mobile Money was piloted in specific regions of each country. In Ghana and Mali, for example, initial buy-in to Mobile Money was limited to specific geographic areas. In Côte d’Ivoire, however, there was nationwide support from the Ministry of Health for the Mobile Money initiative.

Implementation of Mobile Money occurred in 3 phases in each pilot country: precampaign, campaign, and postcampaign. To illustrate the overall implementation process and rollout of Mobile Money, Côte d’Ivoire—the largest and first pilot program—is described. Although there were some minor differences in implementation across countries (e.g., customization of campaign health worker databases in Ghana and Mali to promote advocacy at the local level), the Mobile Money implementation phases were the same for all 3 pilot countries.

**Fig. 1:**
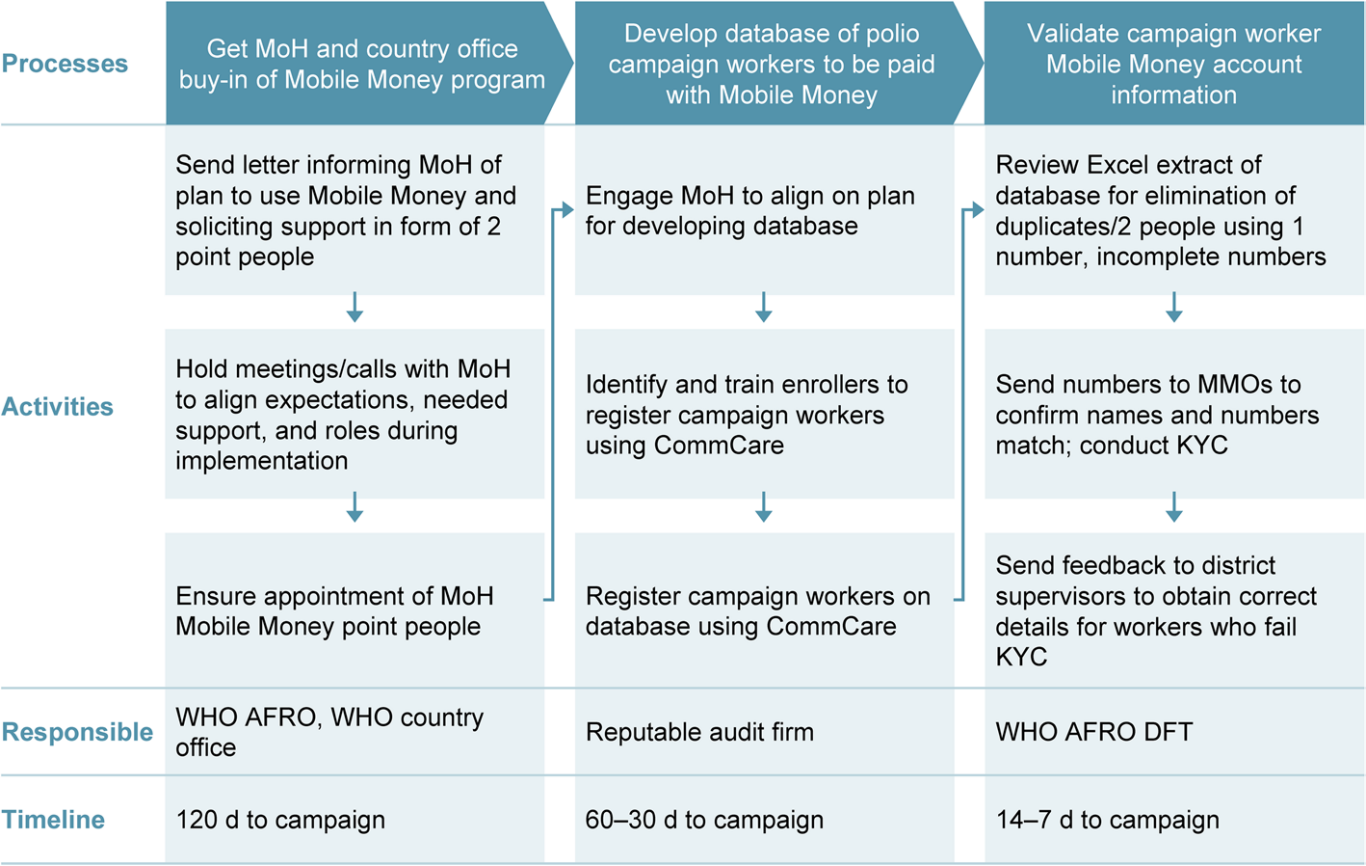
Precampaign phase: Côte d’Ivoire. AFRO, Regional Office for Africa; DFT, Digital Finance Team; KYC, Know Your Customer; MMOs, Mobile Money operators MoH, Ministry of Health; WHO, World Health Organization

Precampaign in Côte d’Ivoire, the AFRO DFT worked in close coordination with the country office to obtain buy-in for the Mobile Money program from the Ministry of Health (Fig. 1). The DFT also initiated efforts to form a national task force comprising all project stakeholders to ensure efficient implementation of digital payments. To develop a database of polio health campaign workers to be paid with Mobile Money, the audit firm PwC was engaged.

At this time, a polio health campaign worker database was developed using the CommCare application (Dimagi, Inc., Cambridge, Massachusetts, USA). Contracts were set up with the Mobile Money operators (MMOs) Orange Money SA (Abidjan, Côte d’Ivoire) and the MTN Mobile Financial Group (Abidjan), who provided interfaces (e.g., wallets, web applications, and agent networks) through which campaign workers could receive payments, withdraw money, and execute other financial functions. At this stage, worker data collected in the field via Commcare were sent to the MMOs for Know Your Customer (KYC) validation to ensure the correct workers were being paid. (KYC guidelines in financial services require professionals to make an effort to verify the identity of their customers and any associated risks with the customer relationship.)

**Fig. 2:**
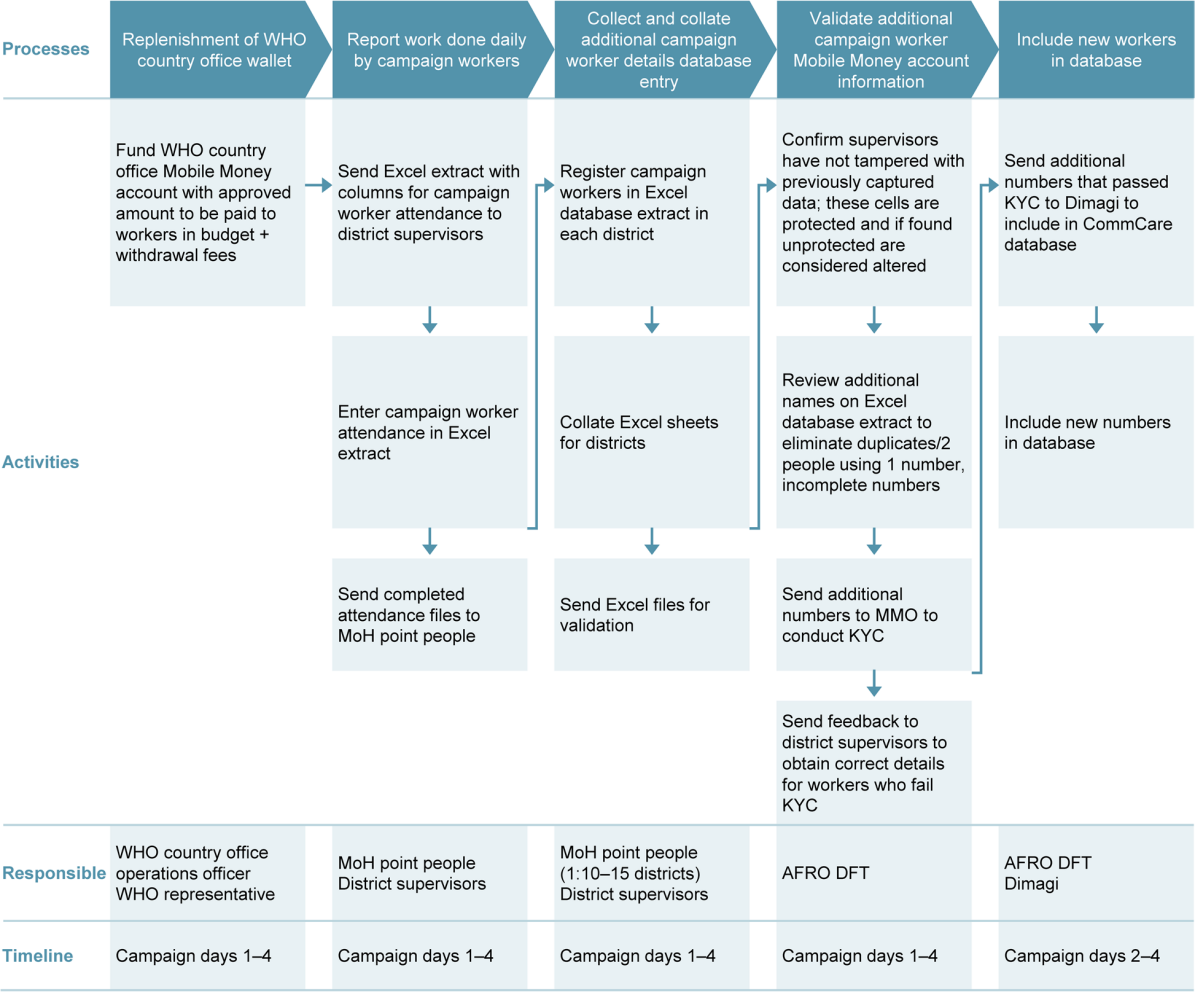
Campaign phase: Côte d‘Ivoire. AFRO, Regional Office for Africa; DFT, Digital Finance Team; KYC, Know Your Customer; MMO, Mobile Money operator; MoH, Ministry of Health; WHO, World Health Organization

During the campaign (Fig. 2) in Côte d’Ivoire, ministry of health point people and district supervisors reported work performed daily by campaign workers. They also collected and collated additional polio health campaign worker details for database entry. The WHO AFRO DFT validated additional campaign worker Mobile Money account information and, along with Dimagi, added new workers to the database.

**Fig. 3:**
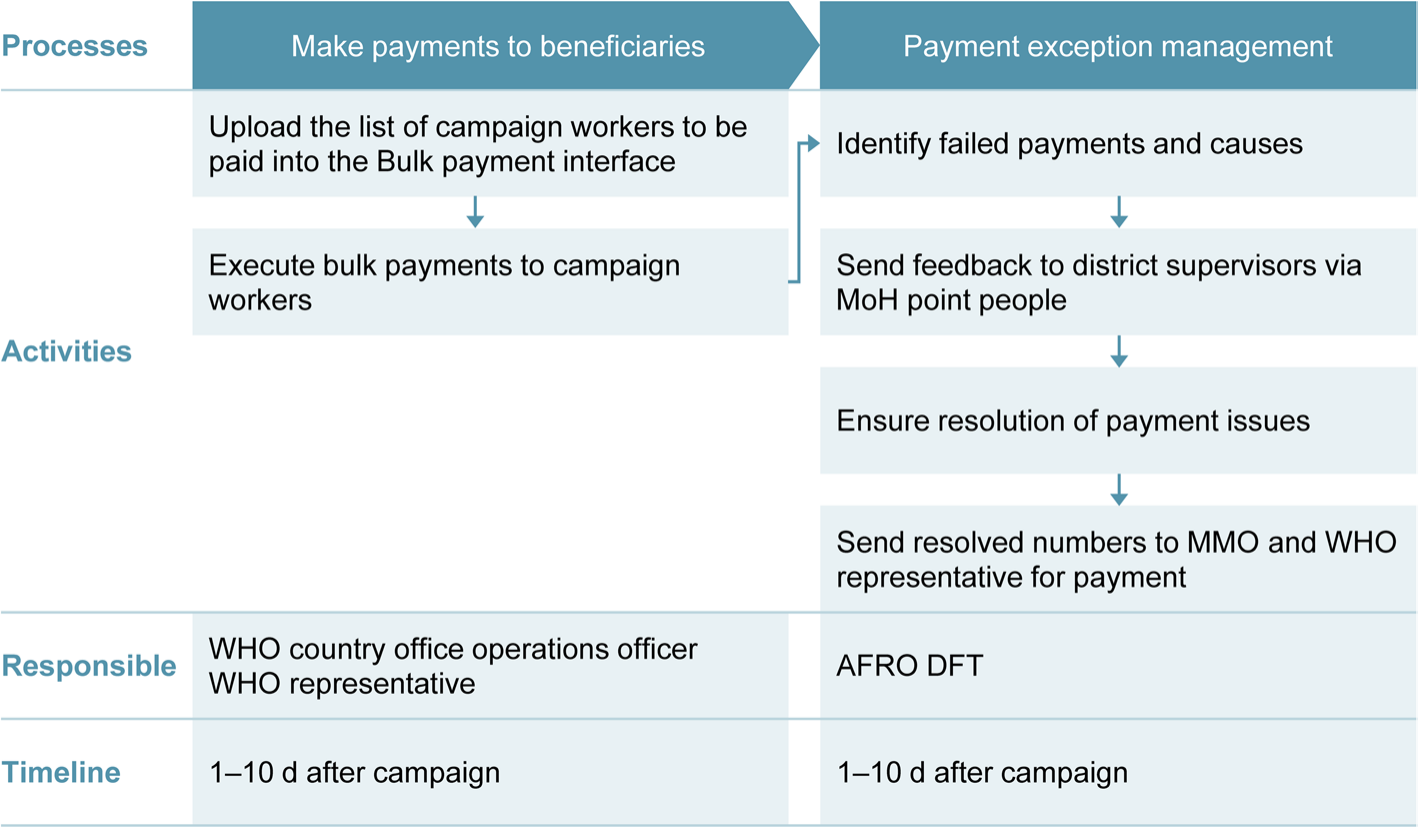
Postcampaign phase: Côte d’Ivoire. AFRO, Regional Office for Africa; DFT, Digital Finance Team; MMO, Mobile Money operator; MoH, Ministry of Health; WHO, World Health Organization

In the Côte d’Ivoire postcampaign (Fig. 3), the WHO Country Office Operations Officer and a WHO representative made payments to polio health campaign workers. The WHO AFRO DFT tracked the success of payments, and conducted account reconciliations to track and resolve payment discrepancies. A postcampaign survey was conducted after Mobile Money program implementation.

## Results

### Number of polio health campaign workers paid via Mobile Money by country

**Fig. 4:**
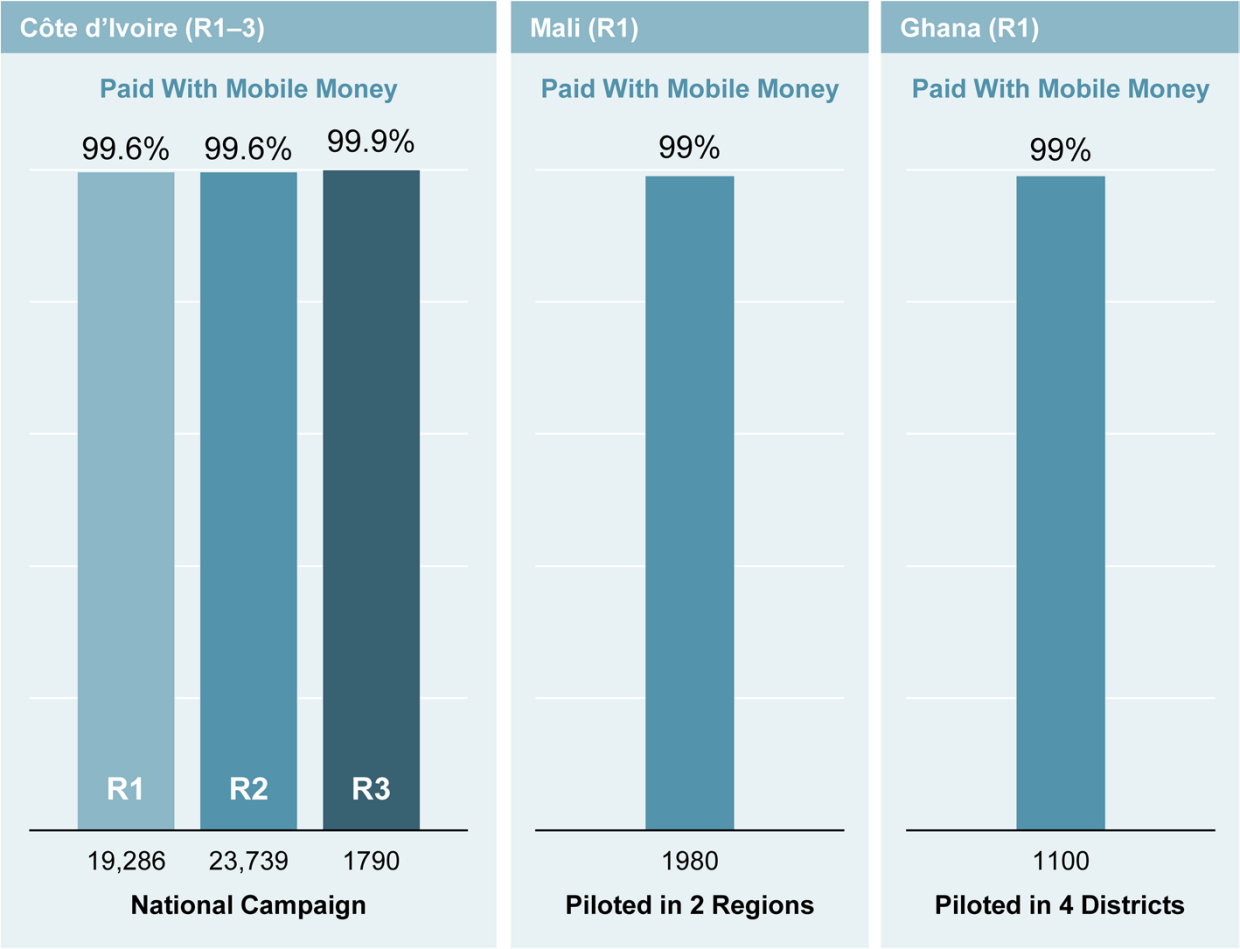
Percentages of polio campaign health workers successfully paid via Mobile Money. R, round

The reach of Mobile Money, as measured by the number of polio health campaign workers enrolled in country-specific databases across the nationwide pilot in Côte d’Ivoire, and the smaller pilots in Ghana and Mali varied (Table 1). The percentages of payments successfully made via Mobile Money in pilot countries were high (Fig. 4). Relative to the national effort in Côte d’Ivoire, the pilot campaigns in 5 districts in Ghana and 2 regions in Mali reached significantly fewer workers. In campaign round 1, however, 99% of health campaign workers in Ghana and 99% in Mali were paid successfully via Mobile Money. In Ghana, any failures resulted from situations in which workers did not have Mobile Money accounts. Notably, Ghana’s pilot program also showed that Mobile Money can successfully reach polio health campaign workers in remote areas. In Mali, 100% (26,000) of workers received payments after completion of the polio vaccination campaigns via Mobile Money within 6 days.

**Table 1:** Overview of Mobile Money pilot country reach

Country	Geographical payment area		Total workers targeted	Workers paid via Mobile Money/time to pay 90% of workers
Côte d’Ivoire	R1 R2 R3	Countrywide	R1: 19,286 R2: 23,739 R3: 1,790	R1: 99.6%/11 d R2: 99.6%/5 d R3: 99.9%/1 d
Mali	R1: 2 of 10 regions R2: 7 of 10 regions		R1: 2000 R2: 26,000	R1: 99%/6 d R2: 100%/5 d
Ghana	R1: 5 of 228 districts		R1: 1100	R1: 99%/7 d

*R* Round

### Côte d’Ivoire successfully implemented Mobile Money for 3 campaign rounds

The Côte d’Ivoire Ministry of Health developed a polio health campaign worker database using CommCare, which captured 44,980 workers. The initial database was developed in 23 days and captured 37,000 polio health campaign workers. The database currently includes 45,000 workers from the 3 polio campaign rounds. The Ministry of Health piloted Mobile Money in all 113 districts for campaign rounds 1 and 2, and in 4 districts in Abidjan for campaign round 3. In rounds 1, 2 and 3, 99.6%, 99.6%, and 99.9% of payments, respectively, were made successfully to workers.

In October and December 2020, 44,815 polio campaign workers received payment within 11, 5, and 1 day for rounds 1, 2, and 3, respectively. To report campaign activities for 95 districts, 14,318 campaign worker attendance sheets were received in round 1. According to the results survey that captured data from 182 workers, 96% wanted to receive subsequent payment via Mobile Money.

### Timing of payments affected worker experience in Cote d’Ivoire

A stratified random sample of polio health campaign workers from all 113 districts in Côte d’Ivoire received the 60 Decibels survey about their experience with Mobile Money, which were completed ~ 4 weeks after the campaign. Of the 514 workers contacted by phone, 382 (74%) responded to questions about the timeliness, completeness, and convenience of Mobile Money payments.

**Fig. 5:**
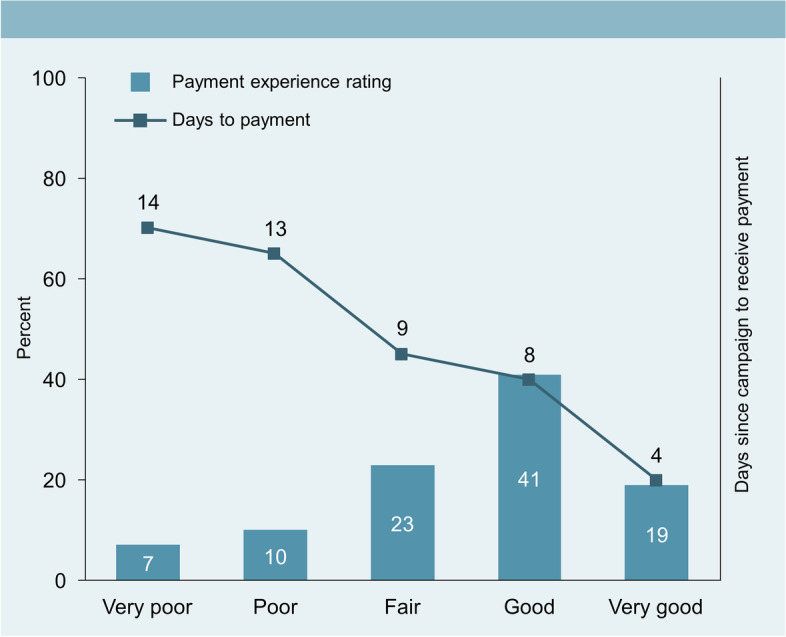
Average payment experience rating and median days taken to receive payment (*n* = 351 for Round 1; *n *= 334 for Round 2)

Among those surveyed, timely payment was associated with positive ratings of the Mobile Money payment system by health campaign workers. Of those who received payments a median of 4 and 8 days postcampaign, 19% and 41% rated their payment experience as “very good” and “good,” respectively (Fig. 5). Conversely, when median time from postcampaign to payment was ~ 2 weeks, workers rated their payment experience as “poor” or “very poor.”

As noted, in several pilot programs, electronic payment delays stemmed from the KYC safeguard system used to verify polio health campaign worker identity. In Mali, for example, the KYC process needed to be repeated to verify worker identity, delaying validation of KYC checks. Payment delays also resulted from the high worker turnover associated with health campaigns. High turnover required ministries of health to identify workers close to campaign start dates, which did not allow sufficient time to secure information necessary for validation and issuance of timely electronic payments. Finally, delayed payments occurred when ministries did not promptly return worker attendance registers to WHO country offices for validation and payment.

### The majority of campaign workers in Cote d’Ivoire were comfortable with Mobile Money payments

Asked whether they preferred cash payments or Mobile Money payments, 83% of polio health campaign workers (*n* = 366) chose Mobile Money. Mobile Money was considered “more secure than cash” by 36% of those surveyed and “easier/more convenient than cash” by 26%. More respondents also felt that Mobile Money was a faster form of payment than cash.

Health campaign workers reported relatively high levels of comfort using Mobile Money independently, along with confidence about their ability to cash out payments when needed. When asked how often they required help using their Mobile Money account, 72% said “never” and 19% said “rarely.” Among this same group of workers, 93% were “very confident” or “somewhat confident” that they could get all the cash in their account when needed.

## Conclusion

### Experience-based lessons can inform future Mobile Money program rollouts

This experience has shown that Mobile Money programs can be successfully implemented on a pilot basis in the WHO AFRO. Some challenges to implementation of a Mobile Money pilot program, however, require long-term structural improvements in technology and greater countrywide mobile access. For example, in DRC, lack of electricity and Internet limited the ability to enroll polio health campaign workers, and in the Republic of the Congo, poor mobile coverage prevented the health facility in 1 district from gaining access to Mobile Money. Even in the absence of sweeping infrastructure development, however, Mobile Money pilot programs in WHO AFRO can help guide successful implementation of digital payment systems across the continent.

The pilot programs revealed that early country and local government engagement in the transition to a digital payment system is critical, and efforts to secure government buy-in and advocacy are needed. In Côte d’Ivoire and DRC, the WHO AFRO DFT started advocacy for Mobile Money with the respective ministries of health as early as database design, which encouraged country ownership of Mobile Money implementation. By positioning the ministry of health to lead Mobile Money activities and provide necessary technical support throughout program implementation, the ministry could build and sustain Mobile Money program advocacy and buy-in among government officials.

The importance of applying a thorough, detail-oriented approach across all aspects of Mobile Money implementation was another key learning from the pilot experiences. In several instances, contract details hampered pilot program rollouts. In Ghana, for example, approval to conduct postcampaign surveys was delayed as a result of an undefined clause in the signed contract. Another detail created an unforeseen situation in Mali: CommCare could not be used to register health campaign workers because data had to be stored outside the country, which is against the data protection policy. By developing and scrutinizing Mobile Money program contracts well in advance of program implementation, teams can ensure that contracts adhere to country guidelines, saving time and effort later.

This proactive, early identification of potential hurdles to program success also applies to relationships with MMOs. In several pilot programs, electronic payment delays were traced back to the KYC safeguard system, which is used to verify polio health campaign worker identity. Specific challenges varied: In Côte d’Ivoire, country regulations prevented MMOs from sharing data within the time frame stipulated in the MMOs’ service-level agreement. In DRC, subscriber identity module cards in phones are not necessarily registered to a specific user, whereas a Mobile Money account is linked to an individual, creating unique issues for KYC verification. Finally, in Mali, there was a need to repeat the KYC process to verify identity and, as a result, validation of KYC checks took longer than anticipated. These incidents point to the value of identifying MMOs and reviewing their services at the precampaign phase to flag potential service-level or technical difficulties.

Pilot program implementation revealed that administrative activities (e.g., data entry, recording worker attendance, and overseeing payments) underly program success and thus assessments of staff capacity or suitable alternatives are critical. In Côte d’Ivoire, CommCare could not be used for attendance reporting due to concerns that information would be input incorrectly as limited staff had familiarity with using the app. At DRC sites, mobilization and allocation of campaign enrollers and supervisors was inefficient due to the need for transportation to site and health zones across many villages; further, some enrollers were found to be unqualified to carry out the enrolment process because they could not read or write in French, or lacked basic knowledge on use of Android devices. In Mali, only 2 people were in charge of payment and administrative activities, which limited overall program outreach and capacity.

Finally, successful program expansion in new regions and countries requires clear documentation of lessons learned in pilot countries. Already, protocols developed based on Mobile Money experiences in Côte d‘Ivoire have guided program design and implementation in DRC and Republic of the Congo. Ideally, a working lessons-learned agenda for the Mobile Money program would: (1) provide evidence that building advocacy among country government decision makers directly helps support the implementation of Mobile Money; and (2) inform the design and implementation of similar digital payment system interventions.

### Current progress and future directions

The scale of this WHO-led project marks a significant innovation in payment efficiency across polio campaigns. Since the initial pilot program, Mobile Money implementation is underway in Burkina Faso, DRC, Gambia, Liberia, Republic of the Congo, Sierra Leone, and South Sudan—and is scheduled to roll out in a total of 47 WHO AFRO countries. The Mobile Money program paves the way not only for improving polio eradication efforts, but also leads the way for other agencies committed to delivering greater efficiencies and smart program implementation throughout the AFRO.

As demonstrated by the WHO Mobile Money pilot program experiences, particularly those in Côte d’Ivoire, transition to Mobile Money has the power to effect real change by providing greater transparency of financial records, improving financial reporting, and decreasing leakages of funds. In turn, individual and regional access to financial applications can expand, and individuals are empowered with opportunities for greater financial resilience and security. This electronic-based payment system promotes timely, direct payments to frontline workers, thereby addressing a key factor fundamental to the strength and viability of vaccination campaigns across health systems.

## Data Availability

The data that support the findings of this study are available from the ministries of health of the countries involved, but restrictions apply to the availability of these data, which were used under license for the present study and thus are not publicly available. The full analyses used for this study are available on the 60 Decibels website repository for Cote d’Ivoire (https://60decibels.com/user/pages/07.Work/_polio_vaccinators/Insights%20From%20Surveying%20Polio%20Vaccinators%20Cote%20dIvoire.pdf), Liberia (https://60decibels.com/user/pages/07.Work/_polio_vaccinators/Insights%20From%20Surveying%20Polio%20Vaccinators%20Liberia.pdf), and Republic of Congo (https://60decibels.com/user/pages/07.Work/_polio_vaccinators/Insights%20From%20Surveying%20Polio%20Vaccinators%20RepublicOfTheCongo.pdf).

## Abbreviations

AFRO: Regional Office for Africa
cVDVP2: Circulating vaccine-derived poliovirus type 2
DFT: Digital Finance Team
DRC: Democratic Republic of the Congo
GPEI: Global Polio Eradication Initiative
KYC: Know Your Customer
MNOs: Mobile Network Operators
RRT: Rapid response team
WHO: World Health Organization
